# Identification of a Kdn biosynthesis pathway in the haptophyte *Prymnesium parvum* suggests widespread sialic acid biosynthesis among microalgae

**DOI:** 10.1074/jbc.RA118.004921

**Published:** 2018-08-31

**Authors:** Ben A. Wagstaff, Martin Rejzek, Robert A. Field

**Affiliations:** From the Department of Biological Chemistry, John Innes Centre, Norwich Research Park, Norwich NR4 7UH, United Kingdom

**Keywords:** algae, sialic acid, carbohydrate biosynthesis, bioinformatics, carbohydrate processing, enzyme, glycobiology, KDN, Prymnesium parvum

## Abstract

Sialic acids are a family of more than 50 structurally distinct acidic sugars on the surface of all vertebrate cells where they terminate glycan chains and are exposed to many interactions with the surrounding environment. In particular, sialic acids play important roles in cell–cell and host–pathogen interactions. The sialic acids or related nonulosonic acids have been observed in Deuterostome lineages, Eubacteria, and Archaea but are notably absent from plants. However, the structurally related C8 acidic sugar 3-deoxy-d-*manno*-2-octulosonic acid (Kdo) is present in Gram-negative bacteria and plants as a component of bacterial lipopolysaccharide and pectic rhamnogalacturonan II in the plant cell wall. Until recently, sialic acids were not thought to occur in algae, but as in plants, Kdo has been observed in algae. Here, we report the *de novo* biosynthesis of the deaminated sialic acid, 3-deoxy-d-*glycero*-d-*galacto*-2-nonulosonic acid (Kdn), in the toxin-producing microalga *Prymnesium parvum*. Using biochemical methods, we show that this alga contains CMP–Kdn and identified and recombinantly expressed the *P. parvum* genes encoding Kdn-9-P synthetase and CMP–Kdn synthetase enzymes that convert mannose-6-P to CMP–Kdn. Bioinformatics analysis revealed sequences related to those of the two *P. parvum* enzymes, suggesting that sialic acid biosynthesis is likely more widespread among microalgae than previously thought and that this acidic sugar may play a role in host–pathogen interactions involving microalgae. Our findings provide evidence that *P. parvum* has the biosynthetic machinery for *de novo* production of the deaminated sialic acid Kdn and that sialic acid biosynthesis may be common among microalgae.

## Introduction

**Figure 1.**
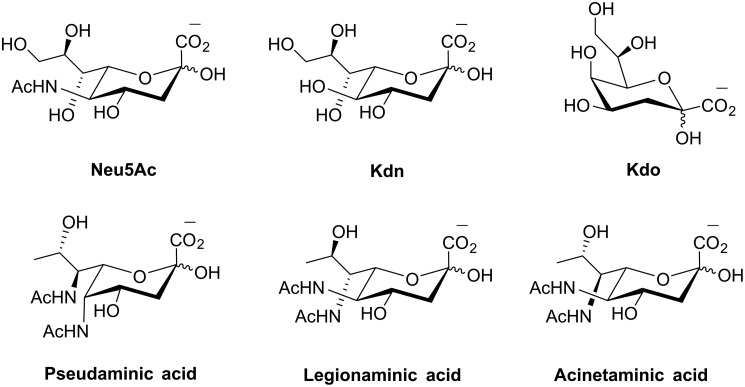
**Structures of C-9 sialic acids and structurally related C-8 acidic sugar, Kdo.**

Sialic acids are acidic nine-carbon carbohydrates found on the surface of all vertebrate cells (1–3). Frequently, sialic acids occupy the terminal position on a glycan, exposing cell-surface sialic acids to a whole range of host–pathogen interactions (4). These interactions are exploited through protein–ligand interactions, where the proteins in question are the well studied family of sialic acid-binding lectins (5), and the sialic acids are the target ligand. Opportunistic pathogens, such as *Escherichia coli* K1 or the influenza virus, often exploit this common biological interaction, where the presentation of host sialic acids (molecular mimicry) can be used to evade host immunity or recognition of host sialic acids can be used to promote virus binding as the first step in infection (6, 7). Alternatively, sialic acids can be modified with chemical moieties such as acetyl groups to evade acquired immunity, and this has led to the discovery of over 50 different naturally occurring sialic acid variants (3). The most common sialic acid in humans is Neu5Ac, first discovered by Klenk and Blix in the 1930s (8). However, other types of nine-carbon sialic acids exist in bacteria, such as the di-*N*-acetylated neuraminic acids, pseudaminic acid, legionaminic acid, and acinetaminic acid (9), as well as the deaminated neuraminic acid, 3-deoxy-d-*glycero*-d-*galacto*-2-nonulosonic acid (Kdn)^2^ (Fig. 1) (10).

Kdn was first discovered by Nadano *et al.* (10) in 1986 in rainbow trout eggs and has since been observed in salmon (11) and amphibian eggs (12–14), other fish organs (15), and pathogenic bacteria (16, 17). Its presence has also been observed at varying levels in different mammalian tissues and cancer cells (18, 19), sometimes as a result of promiscuous Neu5Ac biosynthesis (20). More recently, reports of Kdn biosynthesis have been observed in the human gut symbiont *Bacteroides thetaiotaomicron* (21), and the first report of sialic acids in microalgae is that of a Kdn-linked sphingolipid in the lipid rafts of the haptophyte *Emiliania huxleyi* (22, 23).

In connection with our work on the devastating impact of harmful algal blooms in the waterways of the East of England (*e.g.* the Norfolk Broads) (24–26), we have cause to investigate sialic acids in the toxin-producing haptophyte *Prymnesium parvum*. The haptophytes are a widespread division of microalgae that play crucial roles in the oceanic carbon and sulfur cycles (27, 28); they also form blooms that are toxic to fish (29, 30). The toxin-producing species in this family mainly belong to the *Prymnesium* and *Chrysochromulina* genera, of which the most studied species is *P. parvum*, which is known to cause harmful blooms that result in mass fish mortalities because of the release of natural product toxins (23–34). *P. parvum* is cosmopolitan, with blooms causing economic disaster on all continents except Antarctica (35). In evolutionary terms, the haptophyte family are derived from the red algal plastid lineage, which has undergone significant incoming horizontal gene transfer from bacteria (36). This is supported by a *P. parvum* transcriptome from the Marine Microbial Eukaryote Transcriptome Sequencing Project (MMETSP) (37), which contains a plethora of both eukaryotic and prokaryotic genes, which may aid the alga in allelopathy during nutrient limitation when competition is at its highest. Recent work from our group has discovered a novel lytic megavirus that infects *P. parvum* and has been implicated in the crash of toxic blooms of this species (25).

Research into algal viruses has taken off in the last two decades, prompted by the discovery of the *Acanthamoeba polyphaga* mimivirus in 2003 (38). Algal viruses have since been shown to play crucial roles in ecosystem dynamics (39) and biogeochemical cycles (40), where large scale lysis of algal populations can lead to changes in waterway nutrient cycles. Although the effect of algal viruses has been studied in some detail, the molecular mechanisms underlying microalgal infection and lysis remain poorly understood. It has been suggested that algal viruses utilize mammalian virus infection strategies, hijacking autophagy pathways (41) and potentially utilizing host cell-surface sialic acids to facilitate infection (22, 23).

**Figure 2.**
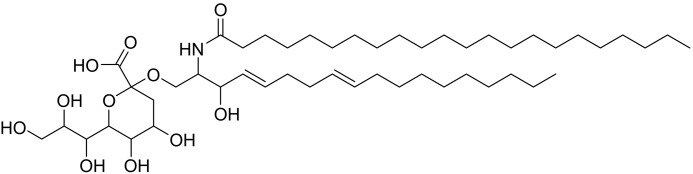
**Putative structure of a Kdn-containing sphingolipid, isolated from the haptophyte *E. huxleyi* (22).** Absolute and relative stereochemistry was not defined.

Recent reports of a Kdn-containing sphingolipid in *E. huxleyi* (22, 23) (Fig. 2), the devastating effect of the phylogenetically related *P. parvum* on fish aquaculture (30), and the large genetic capacity of the haptophyte family made us question whether *P. parvum* was capable of *de novo* sialic acid biosynthesis. Herein, we report the bioinformatics-guided discovery of a biosynthetic pathway leading to CMP-activated Kdn, as well as showing that multiple strains of the *Prymnesium* genus contain Kdn. We also show that the nucleotide sugar, CMP–Kdn, accumulates to significant levels in *P. parvum* cells. In addition, using phylogenetic analysis we show the capacity for widespread sialic acid biosynthesis among the haptophytes and dinoflagellates, members of the Alveolata phyla. Extending these analyses, we show that biosynthetic capabilities for the structurally similar 3-deoxy-d-*manno*-2-octulosonic acid, Kdo, are found scattered across algal groups. This work conclusively shows sialic acid biosynthesis in a unicellular eukaryote microalga, *P. parvum*, and suggests that the occurrence of this metabolic pathway in unicellular eukaryotes is widespread.

## Results

### DMB–HPLC analysis of sialic acids

**Figure 3.**
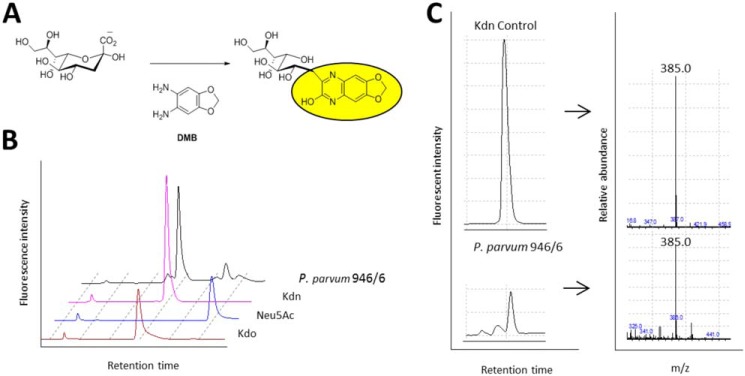
***Prymnesium* spp. contain the deaminated sialic acid Kdn.** *A*, general scheme for DMB labeling of Kdn. The *yellow oval* represents a fluorescent product. *B*, *brown*, Kdo standard; *blue*, Neu5Ac standard; *pink*, Kdn standard. *P. parvum* 946/6 contains peaks aligning with Kdn/Kdo and are confirmed to be Kdn by subsequent *m*/*z* analysis (*C*). *C*, *P. parvum* (Culture Collection of Algae and Protozoa 946/6) contains a peak coeluting with Kdn that shows the expected pseudomolecular ion *m*/*z* 385 of the derivatized Kdn.

To determine the presence and type of sialic acid(s) in species of the *Prymnesium* genus, 15 strains were screened. Each strain was subjected to mild acid hydrolysis to release free sialic acids. Following removal of insoluble cell debris, the resulting supernatants were derivatized with 1,2-diamino-4,5-methylenedioxybenzene (DMB) (Fig. 3*A*) (42) and analyzed by LC–MS with in-line fluorescence detection. Peaks aligning with the standard Neu5Ac were not found (Fig. 3*B*), although peaks aligning with the authentic standard Kdn could be observed in all *P. parvum* 946/6 (Fig. 3*B*) and 14 other strains (Fig. S1*B*); these data aligned well with reported literature values for DMB–Kdn adducts (43). The prospective Kdn peaks also showed masses identical to those seen for the Kdn standard (Fig. 3*C*). Minor peaks aligning with the Neu5Ac standard could be observed (Fig. 3*B*), but upon manual inspection masses corresponding to DMB-Neu5Ac and the DMB-adducts of other sialic acids could not be observed in any of the strains examined (43). Taken together, these results confirm that *Prymnesium* strains produce the deaminated sialic acid Kdn.

### Sugar nucleotide profiling of CMP–Kdn

Monosaccharides are frequently activated inside cells by attachment to nucleoside mono- or diphosphates (44); in the case of sialic acids, they are activated as CMP adducts (3). Therefore, we next sought to establish the presence and levels of intracellular CMP–Kdn in *P. parvum* 946/6. Whole cells were extracted using ethanol in late-log phase, using a modification of a method published by Turnock and Ferguson (45). The extracts were then dried and defatted by partitioning between water and butan-1-ol. Finally, the samples were subjected to solid phase extraction using EnviCarb graphitized carbon columns following previously established methods (46). Based on work by Pabst *et al.* (47), intracellular levels of CMP–Kdn were quantified using LC–MS/MS. A detailed protocol for the extraction and quantification of sugar nucleotides can be found in the work of Rejzek *et al.* (44).

CMP–Kdn produced enzymatically in this study was used as an authentic standard to generate multiple reaction monitoring transitions and to determine retention times of the analyte. Coinjection of cell extracts with the authentic standard was used to confirm analyte identification. Using this methodology, a strong signal for CMP–Kdn was observed in extracts of *P. parvum* 946/6 (Fig. S2*A*). Using peak area integration and an internal standard, GDP-α-d-Glc, levels of intracellular CMP–Kdn were estimated to be 207 ± 72 pmol/g of wet cell pellet (Fig. S2*B*). Concentrations of two other sugar nucleotides used in this study for comparison were found to be 71 ± 17 (UDP-α-d-Gal) and 328 ± 101 (UDP-α-d-Glc) pmol/g of cell pellet, showing that CMP–Kdn is present at appreciable concentration in *P. parvum*.

### Identification of a putative CMP–Kdn biosynthesis pathway in P. parvum

Protein sequences involved in CMP–Kdn biosynthesis in *B. thetaiotaomicron* VPI-5482 (21) were used to identify transcripts from *P. parvum* with high sequence similarity. Subsequently, sequences from the *P. parvum* transcriptome with high similarity to Kdn-9-P synthetase (CAMPEP_0191217894) and CMP–Kdn synthetase (CAMPEP_0191219004) were identified. No sequences were found with high sequence similarity (*E* values ≤ 1E^−05^) to the second enzyme of the pathway, Kdn-9-P phosphatase. Further analysis of the CMP–Kdn synthetase sequence identified a putative transmembrane domain at the C terminus of the protein. No sequence alignments corresponding to transmembrane domains were seen in the Kdn-9-P synthetase sequence.

### Production of recombinant P. parvum Kdn-9-P synthetase (CAMPEP_0191217894) and CMP–Kdn synthetase (CAMPEP_0191219004)

To confirm the enzymatic activity of the putative *P. parvum* Kdn-9-P synthetase and CMP–Kdn synthetase, both recombinant proteins, as well as the previously characterized *B. thetaiotaomicron* Kdn-9-P phosphatase, were produced by heterologous expression in *E. coli*. All sequences were codon-optimized for expression in *E. coli* and synthesized. The sequences were synthesized with In-Fusion^TM^ cloning overhangs so that they could be cloned directly into the pOPINF vector (48) without the need for rounds of PCR amplification. The recombinant plasmids containing all three sequences were transformed into *E. coli* BL21, and expression was induced at 18 °C using 0.4 mm isopropyl 1-thio-β-d-galactopyranoside. Kdn-9-P synthetase expressed at high levels (24 mg/liter of *E. coli*) (Fig. S3*A*), but purified protein showed a tendency to precipitate if left for more than 2 days. This issue was resolved by including DTT (5 mm) into the protein lysis and extraction buffer, as well as diluting purified fractions after affinity chromatography. Although expression of *B. thetaiotaomicron* VPI-5482 Kdn-9-P phosphatase produced mainly insoluble protein under similar conditions, low milligram levels of soluble protein could be purified without issue (Fig. S3*B*). Initial attempts to express full-length *P. parvum* CMP–Kdn synthetase (273 amino acids) in *E. coli* were unsuccessful, resulting in insoluble protein of the expected mass. Further analysis of the sequence using Phobius (49) identified a C-terminal transmembrane domain, which was subsequently truncated using PCR before cloning and expression were reattempted. Truncated CMP–Kdn synthetase (amino acids 1–233) was cloned and expressed and resulted in reasonable levels (4 mg/liter of *E. coli*) of soluble protein of the expected mass as judged by SDS–PAGE analysis (Fig. S3*C*).

### Biochemical characterization of recombinant P. parvum Kdn-9-P synthetase (CAMPEP_0191217894) and CMP–Kdn synthetase (CAMPEP_0191219004)

**Figure 4.**
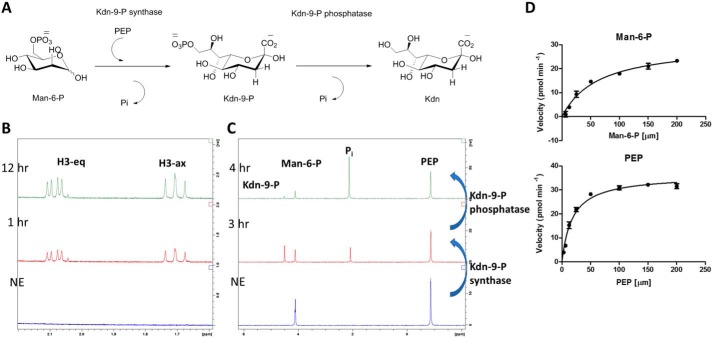
**Biochemical characterization of *P. parvum* Kdn-9-P synthetase.** *A*, reaction catalyzed by Kdn-9-P synthetase and Kdn-9-P phosphatase. *B*, ^1^H NMR analysis following the reaction of Kdn-9-P synthetase. Signals corresponding to equatorial (H3-eq) and axial (H3-ax) protons of Kdn-9-P can be observed 1 and 12 h after Kdn-9-P synthetase enzyme has been added. *C*, ^31^P NMR analysis following the reaction of Kdn-9-P synthetase and Kdn-9-P phosphatase. Before enzyme addition (*NE*), signals corresponding to Man-6-P (∼4.10 ppm) and PEP (−0.87 ppm) can be observed. Following addition of Kdn-9-P synthetase to the reaction mixture for 3 h (3 h), new signals for Kdn-9-P (4.50 ppm) and P_i_ can be observed (2.09 ppm). After addition of Kdn-9-P phosphatase for a further 1 h (4 h), Kdn-9-P has almost entirely been consumed, and additional P_i_ has been formed. *D*, kinetic analysis of Kdn-9-P synthetase with PEP and Man-6-P as substrates. Michaelis–Menten plots were constructed by varying concentrations of one substrate while fixing the other (100 μm).

For biochemical characterization of *P. parvum* Kdn-9-P synthetase, a combination of ^1^H and ^31^P NMR and ESI–MS were used. Based on sequence similarity to *B. thetaiotaomicron* VPI-5482 BT1714 (21), the reaction was anticipated to produce Kdn-9-P from phosphoenolpyruvate (PEP) and Man-6-P. Reaction solutions contained 38 μg of CAMPEP_0191217894, 2 mm MgCl_2_, 8 mm PEP, and 10 mm Man-6-P, buffered in 50 mm HEPES (pD 7.5). Reaction time courses were monitored at room temperature by ^1^H NMR, whereby formation of the axial (H3-ax) and equatorial (H3-eq) protons of the Kdn moiety could be easily followed (21) (Fig. 4, *A* and *B*). For ^31^P NMR, consumption of Man-6-P and PEP could be readily followed (Fig. 4*C*); coinciding with the depletion of these two peaks, formation of two new peaks representing P_i_ and Kdn-9-P could be observed. As with ^1^H NMR values, ^31^P NMR values corresponding to Kdn-9-P aligned well with previously published values (21).

In addition to direct MS and NMR analysis of the reaction product of *P. parvum* Kdn-9-P synthetase, we next tested the reaction product with the previously characterized Kdn-9-P phosphatase from *B. thetaiotaomicron* VPI-5482 (21). A coupled assay was employed with both *P. parvum* Kdn-9-P synthetase and *B. thetaiotaomicron* VPI-5482 Kdn-9-P phosphatase. Kdn-9-P synthetase was added to PEP (in excess) and Man-6-P (10 and 8 mm, respectively) and left for 3 h to produce Kdn-9-P and the reaction by-product, P_i_. Kdn-9-P phosphatase was then added, and the reaction was left for a further 1 h. The consumption of Kdn-9-P relative to the other signals (*i.e.* Man-6-P) and subsequent accumulation of additional P_i_ clearly showed that the product of *P. parvum* Kdn-9-P synthetase is accepted by *B. thetaiotaomicron* VPI-5482 Kdn-9-P phosphatase (Fig. 4*C*).

A colorimetric phosphate-release assay was employed (BioMol Green, Enzo) to establish kinetic parameters for Kdn-9-P synthetase. For determination of *K_m_* values for both PEP and Man-6-P, reactions were set up to include 100 μm of one substrate while varying the concentration of the second. The steady-state kinetic values determined at 25 °C using this method are ^(PEP)^*K_m_* = 18.3 ± 1.8 μm and ^(Man-6-P)^*K_m_* = 63.6 ± 10.5 μm, *k*_cat_ = 14.9 min^−1^ (Fig. 4*D*). This low *k*_cat_ is typical for other enzymes of this family; human NeuNAc-9-P synthase and *B. thetaiotaomicron* Kdn-9-P synthase both have *k*_cat_ values between 0.67 and 1.31 min^−1^ (21, 50).

The sugar specificity of Kdn-9-P synthetase was then examined by screening with a range of alternative sugar substrates known to produce sialic acid products with other related aldolase enzymes (Table 1). Reaction conditions were the same as previously described with PEP concentrations slightly lower than the sugar substrates (8 and 10 mm, respectively) to ensure that PEP consumption could be attributed to complete conversion by ^31^P NMR. ESI–MS of the reaction mixtures after 18 h was carried out to look for the respective sialic acid products. Conversion was only seen when Man-6-P was used as the starting sugar substrate, which had gone to completion at the 18-h time point. Masses attributable to other sialic acid products could not be detected in any reaction mixture, nor was any level of conversion observed by ^31^P NMR, suggesting an absolute preference for Man-6-P and Kdn-9-P production.

**Table 1:** **Specificity of Kdn-9-P synthetase for Man-6-P as determined by ^31^P NMR and ESI–MS of the reaction mixture after 18 h** All sugar substrates tested are in the d-configuration.

Substrate	Predicted product	^31^P NMR conversion at 18 h	ESI–MS detection of product at 18 h
		%	
Man-6-P	Kdn-9-P	100	Y
ManNAc-6-P	Neu5Ac-9-P	0	N
Ara-5-P	Kdo-8-P	0	N
Man	Kdn	0	N
ManNAc	Neu5Ac	0	N
ManN	Neu	0	N
Ara	Kdo	0	N

**Figure 5.**
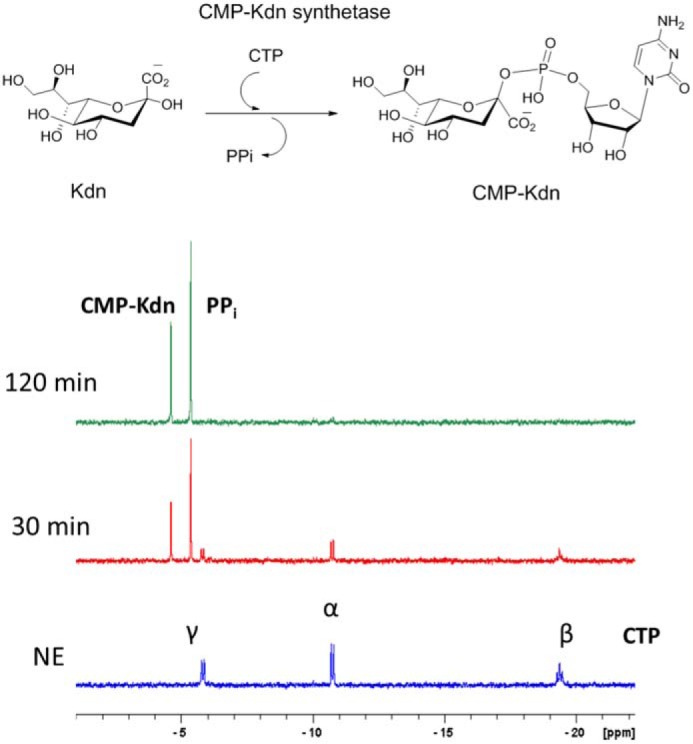
**Reaction catalyzed by *P. parvum* CMP–Kdn synthetase.** Reactions were monitored over 120 min by ^31^P NMR. *Blue* (*NE*) shows three phosphorus signals from the starting material CTP. Over the 120-min time course, two new singlets appear for PPi (−5.37 ppm) and CMP–Kdn (−4.61 ppm). The reaction reaches near completion at this point (∼98% by peak area integration).

Following cleavage of phosphate from Kdn-9-P to produce Kdn, it was speculated that the last enzyme in the pathway would be a CMP–Kdn synthetase to active Kdn. For this reason, CTP and Kdn (or Neu5Ac) were used as substrates in a buffered solution. The reactions were monitored at 25 °C by ^31^P NMR to follow reaction progress (Fig. 5), where loss of signals for CTP and subsequent formation of signals for CMP–Kdn and the by-product PP_i_ could be observed. No conversion was observed by ^31^P NMR or subsequent MS analysis when Neu5Ac was used as a substrate in place of Kdn. When the reaction with Kdn reached completion, the product was purified by strong anion exchange, before being subjected to ^1^H NMR, ^31^P NMR, and ESI–MS analysis. All NMR values aligned well with reported literature values (21), and MS confirmed the expected identity of CMP–Kdn.

### Bioinformatics analysis of sialic acid synthetase and CMP–Kdn synthetase machinery across the algal groups

To identify algal transcripts involved in sialic acid biosynthesis across species, BLASTp searches were carried out using the newly discovered sequences for Kdn-9-P synthetase and CMP–Kdn synthetase from *P. parvum* as search terms. These searches were performed against 153 translated transcriptomes from MMETSP (37) or genomes from NCBI (51), where available. It was quickly noted that 33 algae examined contained sequences with high sequence similarity to Kdo-8-P synthetase, which is distinguishable by sequence from the sialic acid synthetases that produce the C9 sialic acids Kdn and Neu5Ac (52). Therefore, the Kdo-8-P synthetase transcript (CAMPEP_0191499076) and CMP–sugar synthetase transcripts (CAMPEP_0191478336) from one of these algae, *Pyrammimonas parkae*, were also used as consensus sequences to query the nucleic acid databases to ensure disparity between the two sets of sequences. Sialic acid phosphatases were not examined because of the functionally redundant nature of these enzymes (53).

**Figure 6.**
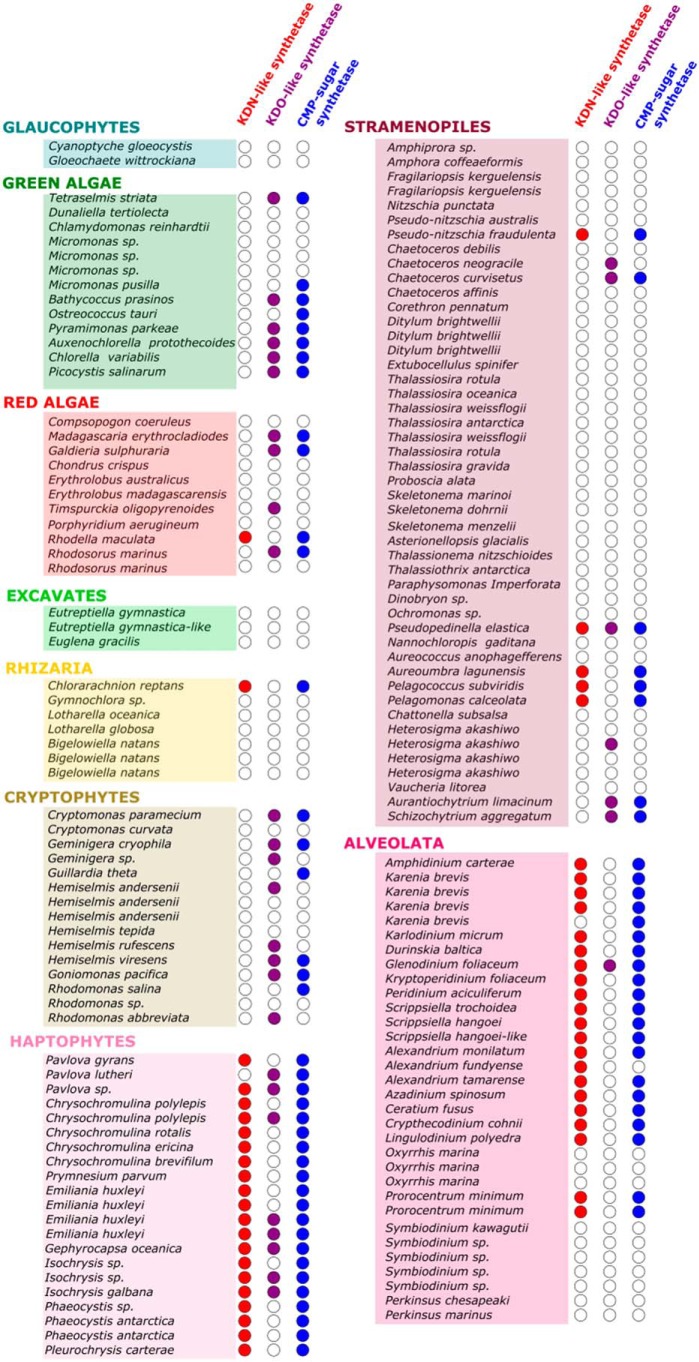
**Table showing the distribution of sialic acid and Kdo biosynthesis pathways in algae.** A total of 153 transcriptomes or genomes were analyzed for the presence of Kdn- and Kdo-like synthetases, as well as the corresponding CMP–sugar synthetase enzymes. Where a transcript was found for a given gene, a *filled circle* can be found. For Kdn-like synthetases, the *circles* are *red*. For Kdo-like synthetases, the *circles* are *purple*. For CMP–sugar synthetase genes, the *circles* are *blue*. Where the same species is mentioned more than once, different strains have been analyzed. A full list of strains used in this study, as well as sources of transcriptome, genome, and sequence identifiers, can be found in supporting Table S2.

As expected, most species of algae that contained sequences with high sequence identity to either the C9-like sialic acid synthetases or C8-like 3-deoxy-d-*manno*-2-octulosonic acid (Kdo) synthetases also encoded CMP–sugar synthetases to active said sugars (Fig. 6). Only 14 of 153 algal strains examined had one of the two parts of the pathway. Sequences involved in sialic acid or Kdo biosynthesis could be found across most algal groups, except the glaucophytes, excavates, and rhizaria, in which only *Chlorarachnion reptans* contained sialic acid biosynthetic machinery (Fig. 6). Of the 153 database entries examined, 48 contained sequences with high sequence similarity to the Kdn-9-P synthetase from *P. parvum*. Of these 48, only 7 are found outside of the Haptophyta and Dinoflagellata phyla, with all but 1 of the 21 haptophytes examined found to contain homologues of this enzyme. Homologues of Kdo-8-P synthetase are more scattered across the algal groups but appear to be particularly frequent in green algae and cryptophytes (6 of 13 and 8 of 15, respectively). Sequences for both Kdo and C9 sialic acid synthesis appear to be mutually exclusive with some exceptions in the haptophytes (*e.g. Isochrysis galbana*) (Fig. 6).

**Figure 7.**
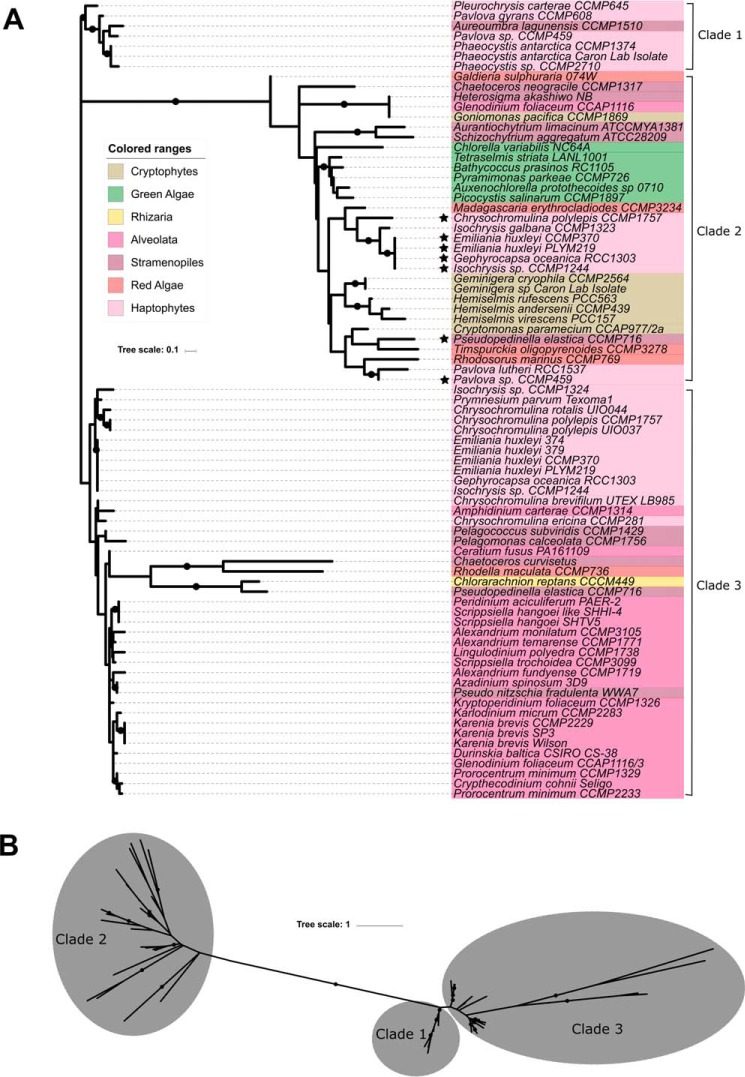
**Phylogenetic analysis of sialic acid and Kdo synthetases across the algal groups.** *A*, phylogenetic analysis of sialic acid and Kdo synthetases. Sequences with high homology to *P. parvum* Kdn-9-P synthetase can be found in clades 1 and 3. Sequences with higher homology to *P. parkae* Kdo-8-P synthetase can be found in clade 2. *B*, unrooted version of tree in *A* showing close relationship of clades 1 and 3. Organisms marked with a *star* appear twice in the tree. Alignments were performed using the default settings of MAFFT (54), and unrooted maximum likelihood phylogenetic trees were produced for sialic acid synthetases (79 sequences). Trees were drawn using MEGA7 (55) and iTOL (56), and the final tree is based on 35 ungapped amino acid positions. Bootstrap values are a result of 100 resampling permutations. Branches with bootstrap support >50% are labeled with a *black circle*. Algal groups are colored roughly according to the scheme in Fig. 6.

**Figure 8.**
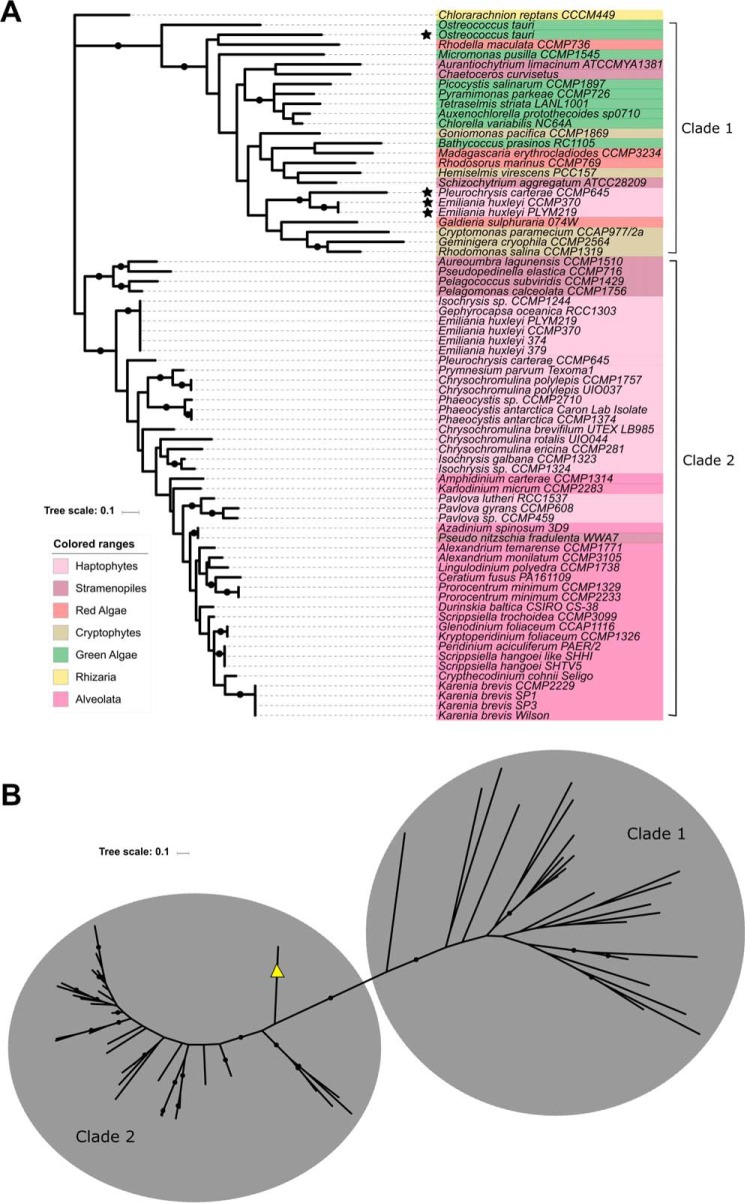
**Phylogenetic analysis of CMP–sugar synthetases across the algal groups.** *A*, phylogenetic analysis of CMP–sugar synthetases. Sequences with higher homology to *P. parkae* CMP–sugar synthetase can be found in clade 1. Sequences with higher homology to *P. parvum* CMP–Kdn synthetase can be found in clade 2. *B*, unrooted version of tree in *A* showing the distinct clades of CMP–sugar synthetases. The sequence from *C. reptans* is marked with a *yellow triangle* and is more closely related to clade 1. Organisms marked with a *star* appear twice in the tree. Alignments were performed using the default settings of MAFFT (54), and unrooted maximum likelihood phylogenetic trees were produced for CMP–sugar synthetases (72 sequences). Trees were drawn using MEGA7 (55) and iTOL (56), and the final tree is based on 66 ungapped amino acid positions. Bootstrap values are a result of 100 resampling permutations. Branches with bootstrap support >50% are labeled with a *black circle*. Algal groups are colored roughly according to the scheme in Fig. 6.

Maximum likelihood phylogenetic trees were constructed to compare sialic acid synthetase/Kdo synthetase genes and CMP–Kdn synthetase genes across the algal groups (Figs. 7 and 8). Fig. 7*A* shows that sequences with higher sequence identity to *P. parvum* Kdn-9-P synthetase (clade 3) do not form a monophyletic lineage with sequences more homologous to Kdo-8-P synthetase-like sequences (clade 2). A small clade containing sequences primarily from the haptophytes (one stramenopile) can also be seen that is more closely related to the Kdn-9-P synthetase sequences in clade 1 than the Kdo-8-P synthetases in clade 2 (Fig. 7*B*). Both clades 1 and 2 with higher homology to Kdn-9-P synthetase from *P. parvum* are dominated by sequences from the Alveolata and Haptophyta, whereas sequences with higher homology to Kdo-8-P synthetase in clade 2 are scattered throughout the algal groups. CMP–Kdn synthetase sequences show a similar trend (Fig. 8), with clade 2 showing higher homology to the CMP–Kdn synthetase from *P. parvum* and clade 1 showing higher homology to the CMP–sugar synthetase sequence from *P. parkae* (CAMPEP_0191478336). As with the sialic acid synthetases, the clade containing sequences with higher homology to CMP–Kdn synthetase from *P. parvum* is dominated by transcripts from the Alveolata and Haptophyta, whereas clade 1 contains a mixture of algae from most groups examined.

## Discussion

**Figure 9.**
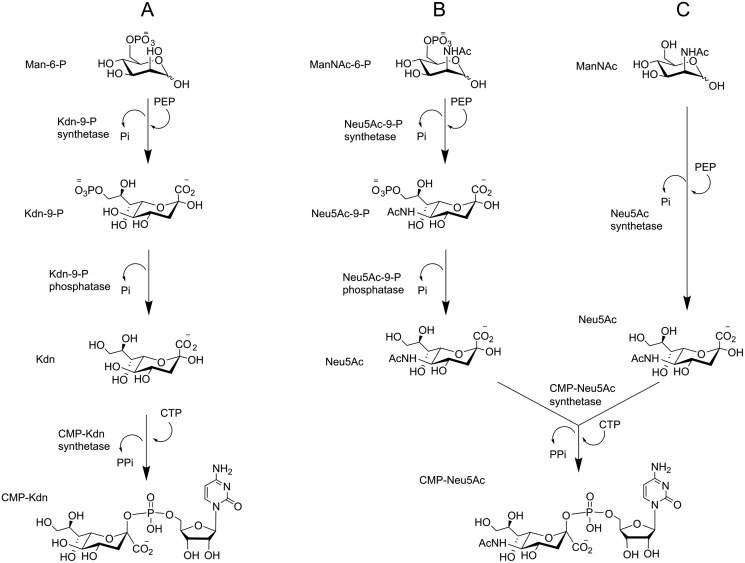
**Biosynthetic pathways of CMP–Neu5Ac in humans and bacteria (*B* and *C*, respectively) and CMP–Kdn in *B. thetaiotaomicron* and *P. parvum* (*A*).** Pathways for sialic acid biosynthesis in *B. thetaiotaomicron*, *P. parvum*, and humans follow phosphorylation/dephosphorylation steps, whereas many bacteria (*C*) do not utilize phosphorylated sugars as the starting point of the pathway and therefore do not need an additional step for phosphate removal.

Although sialic acid biosynthesis has been explored well in bacteria and humans (Fig. 9) (1), reports of sialic acid presence and biosynthesis in algae are very limited and absent, respectively. Here we provide the first biochemical evidence and supporting bioinformatics analysis that the toxin-producing microalga *P. parvum* produces the deaminated sialic acid Kdn *de novo*. LC–MS data highlight the presence of Kdn in 15 different *Prymnesium* strains and also identified CMP–Kdn as the activated form of the sugar. This was supported by the identification of sequences in a publicly available transcriptome for *P. parvum* (MMETSP, Texoma1), which have high sequence identity to known Kdn-9-P synthetase and CMP–Kdn synthetase genes. The corresponding proteins were successfully expressed in *E. coli*, and synthesis of CMP–Kdn from Man-6-P was demonstrated experimentally. Using the validated *P. parvum* sequences, 153 algal nucleic acid databases from MMETSP (37) or NCBI (51) were screened for sialic acid biosynthesis machinery. Sequences with similarity to the Kdn-9-P synthetase from *P. parvum* were abundant among the haptophytes and dinoflagellates, whereas sequences with homology to the structurally related Kdo-8-P synthetase were found scattered across the algal groups (Fig. 6). These bioinformatic findings are consistent with the little that is currently known about sialic acids or Kdo in algae; Kdn has been observed in the haptophyte *E. huxleyi* (22, 23), and Kdo has been observed in the green alga *Tetraselmis striata* (57).

Although a clear homolog of Kdn-9-P phosphatase could not be found in the *P. parvum* transcriptome, the pathway is presumed to follow a phosphorylation/dephosphorylation pathway such as seen in *B. thetaiotaomicron* (21) (Fig. 6) because the Kdn-9-P synthetase does not convert Man to Kdn. This lack of Kdn-9-P phosphatase homolog is not surprising, however; the functional redundancy of sugar phosphatases has been reported previously (53). What is surprising is the micromolar *K_m_* for Man-6-P for Kdn-9-P synthetase (Table 2). This low Michaelis constant suggests that the sialic acid synthetase from *P. parvum* has a much higher affinity for its substrate than other previously studied sialic acid synthetases (Table 2). This may in part be explained by the specificity that *P. parvum* Kdn-9-P synthetase shows for Man-6-P (Table 1), a trait that is not observed in other sialic acid synthetases, which show a level of promiscuity (50). Also notable is the high *k*_cat_ value observed for this enzyme (14.9 min^−1^); most enzymes in this family have *k*_cat_ values closer to 1 min^−1^ (21, 50). In agreement with these findings, CMP–Kdn synthetase from *P. parvum* does not produce CMP–Neu5Ac when Neu5Ac is used as a substrate, suggesting that the key aldolase and synthetase enzymes of this pathway are specific for the production of CMP–Kdn.

**Table 2:** ***K_m_* values for sugar substrates used by Kdn-9-P synthetase in this study and other previously characterized sialic acid synthetase enzymes** All sugar substrates tested are D-configured.

Host organism	Substrate	*K_m_*
		*mm*
*P. parvum* (this study)	Man-6-P	0.064
*B. thetaiotaomicron* (Ref. 21)	Man-6-P	1.4
*Homo sapiens* (Ref. 50)	Man-6-P	2.62
*H. sapiens* (Ref. 50)	ManNAc-6-P	1.04
*Neisseria meningitidis* (Ref. 50)	ManNAc	11.6
*Campylobacter c* (Ref. 58)	ManNAc	17.6

Using the newly discovered sequences from *P. parvum*, 153 nucleic acid databases of other algae were screened for sialic acid or Kdo biosynthetic machinery. Sequences with homology to genes involved in Kdo biosynthesis were found spread across the algal groups, whereas sialic acid synthetase homologs were found to be more confined to the Haptophyta and Alveolata phyla. Although there are exceptions, the biosynthesis of Kdo and sialic acids appears to be mutually exclusive, suggesting that these acidic carbohydrates may complement one another physiologically; indeed, it has previously been suggested that genes involved in sialic acid biosynthesis have been “reinvented” from the Kdo pathway, which share common ancestral genetic origins (3). Some haptophytes contain both sialic acid and Kdo biosynthetic machinery, which may be a result of the extensive endosymbiotic gene transfer that has occurred within the algal groups (59). Phylogenetic trees created to study the evolutionary distance between Kdo/sialic acid synthetases and CMP–sugar synthetases show similar trends. Both trees show two main groups of sequences, showing differences between the Kdo and sialic acid pathways. An additional clade exists for the sialic acid synthetase tree, clade 1 (Fig. 7*A*), which contains almost solely haptophyte sequences. This group is more closely related to the Kdn-9-P synthetase clade 3, which may suggest these sequences are involved in the biosynthesis of alternative C9 sialic acids (3).

The importance of sialic acids in host–pathogen interactions is well documented (60), with a key example being the binding of the human influenza virus to Neu5Ac residues on human epithelial cells (5). More recently, viruses have been shown to play crucial roles in the population dynamics of algae (61), where they influence both ecosystem dynamics (39) and biogeochemical cycles (40). The *E. huxleyi* viruses are probably the best studied of the algal viruses (62), and recently Fulton and co-workers (22, 23) discovered key roles for a novel host sialic acid lipid in viral infection of this alga. Although Kdo had been reported in algae previously, C9 sialic acids were unknown to this group of organisms prior to the Fulton work. Our work now conclusively demonstrates that the toxin-producing microalga *P. parvum* is capable of *de novo* Kdn biosynthesis. The discovery of these new gene sequences was used to show that sialic acid biosynthesis occurs extensively throughout the haptophytes and dinoflagellates. We were also able to highlight the distribution of biosynthetic genes for the structurally related Kdo across the algal groups. The widespread occurrence of these molecules and the ever-increasing number of algal viruses being discovered suggests that sialic acids may have key roles to play in early eukaryote host-pathogen interactions.

## Experimental procedures

### Growth and maintenance of Prymnesium cultures

Cultures of *Prymnesium* were obtained from the Culture Collection of Algae and Protozoa and the culture collection at the Marine Biological Association (Plymouth, United Kingdom). The cultures were grown in f/2-Si medium (7–8 practical salinity units) at 22 °C on a 14:10 light cycle as previously described (25). Stock cultures were made axenic by treatment with carbenicillin (100 μg/ml) as judged by optical microscopy. For metabolite extractions, the cells were harvested at late-log phase of growth which often represented ∼2–3 × 10^6^ cells ml^−1^. A full list of the strains used in this study can be found in the supporting information (Table S1).

### DMB–HPLC analysis of sialic acids

Free sialic acids were liberated from cells by resuspending 10 ml of pelleted *Prymnesium* cells in hydrochloric acid (200 μl, 0.1 m) at 80 °C for 1 h. Insoluble cell debris was then pelleted by centrifugation, and soluble material as well as standards for Kdn (Sigma–Aldrich) and Neu5Ac were then derivatized with DMB per the manufacturer's instructions (sialic acid fluorescence labeling kit; Clontech and Takara Bio Europe SAS, Saint-Germain-en-Laye, France). Control reactions were set up in parallel with HCl to act as a blank background. Derivatized samples (7 μl) were then injected into a Nexera/Prominence ultra high performance liquid chromatography (UHPLC) system (Shimadzu) equipped with an in-line fluorescence detector (excitation, 373 nm; emission, 448 nm) and a LCMS-2020 single quadrupole MS set to scan between 100–600 *m*/*z*. Samples were analyzed on a Kinetex 2.6 μm EVO C18 100 Å LC column (50 × 2.1 mm). Buffer A was an aqueous MeOH, H_2_O buffer (7:93), and buffer B was an organic CH_3_CN, MeOH buffer (93:7). The gradient was set up so that fluorescent DMB-sialic acid adducts eluted within the first 10 mins and was set as follows: 0–10 min 5% B, 10–12.5 min 100% B, 12.5–15 min 5% B, 15–22 min 5% B. The samples were eluted at a flow rate of 0.2 ml min^−1^, and detected peaks were compared with retention times of Kdn, Kdo, and Neu5Ac standards. Previously reported mass values and relative retention times for sialic acids and their *O*-acetyl derivatives were also used for comparison (43).

### Sugar nucleotide profiling of CMP–Kdn

Intracellular sugar nucleotides were extracted and analyzed using previously published methods (44). CMP–Kdn was produced using commercial Kdn (Sigma–Aldrich) and recombinant CMP–Kdn synthetase produced in this study to act as a standard (see “Biochemical analyses” for further information). Other sugar nucleotides used in this study for comparison were UDP-α-d-Glc, UDP-α-d-Gal, and GDP-α-d-Glc as an internal standard for quantification. An authentic standard of CMP–Kdn was used to determine relative retention time and to generate multiple reaction monitoring transitions (Fig. S2). Coinjection of cell extracts with CMP–Kdn standard confirmed identity of peaks.

### Identification of putative CMP–Kdn biosynthesis transcripts in P. parvum

For the identification of transcripts involved in sialic acid biosynthesis from *P. parvum*, BLASTp (63) analysis was carried out against a publicly available transcriptome of *P. parvum* (Texoma1, MMETSP (37)). Protein sequences involved in CMP–Kdn biosynthesis from *B. thetaiotaomicron* VPI-5482 (21) were used as consensus sequences (ExPASy accession numbers Q8A712, Q8A711, and Q8A710). Any hits with *E* values ≤ 1E^−10^ were then analyzed manually for conserved domains before being assigned as a hit. Hits were subsequently analyzed for transmembrane domains and signal peptides using the online tool Phobius (49) (http://phobius.sbc.su.se/).^3^

### Recombinant protein production

Transcripts identified with high sequence similarity to Kdn-9-P synthetase (CAMPEP_0191217894) and CMP–Kdn synthetase (CAMPEP_0191219004), along with Kdn-9-P phosphatase from *B. thetaiotaomicron* VPI-5482 (ExPASy accession number Q8A712), were codon-optimized for expression in *E. coli* using IDTdna's codon optimization software (https://www.idtdna.com/CodonOpt).^3^ The resulting sequences were then synthesized with overhangs for In-Fusion^TM^ cloning into pOPINF vector (48) using IDT's gBlock gene fragment synthesis service. A full list of sequences used in this study can be found in the supporting information.

gBlock fragments were then cloned into pOPINF vectors using In-Fusion^TM^ cloning kit (Clontech) per the manufacturer's instructions. The resulting plasmids were then transformed into Stellar competent cells, before being propagated and extracted (miniprep kit; Qiagen). Positively transformed plasmids were identified by size comparison to a nontransformed pOPINF control using gel electrophoresis. Plasmids containing the gBlock sequences were then finally transformed into BL21 CodonPlus^TM^ (Agilent Technologies and Fisher Scientific) for protein expression. For all proteins, 1 liter of *E. coli* cells were grown to an OD of 0.6 at 37 °C before being transferred to 18 °C for 1 h. Induction was performed with 0.4 mm of isopropyl β-d-thiogalactopyranoside, and the cells were left at 18 °C overnight. Proteins were extracted in the following buffer: 50 mm Tris-HCl, pH 7.5, 0.5 m NaCl, 20 mm imidazole, protease inhibitor mixture (Sigma) 1/100 (v/v), 2 mg of DNase. Kdn-9-P synthetase also required addition of 5 mm DTT to prevent precipitation of the enzyme. All proteins were purified using nickel affinity chromatography, and fractions judged to be >95% pure by SDS–PAGE were pooled for further analysis.

### Biochemical analyses

For Kdn-9-P synthetase, reaction mixtures contained 38 μg of CAMPEP_0191217894, 2 mm MgCl_2_, 8 mm of phosphoenolpyruvate, and 10 mm of Man-6-P, buffered in 50 mm HEPES (pD 7.5). A no-enzyme control time point was taken prior to addition of enzyme. Once enzymes were added, the time points were recorded for ^1^H and ^31^P NMR. ^1^H NMR signals representing H3-eq (∼2.1 ppm) and H3-ax (∼1.7 ppm) were monitored over the time course for formation of Kdn-9-phosphate. ^31^P NMR was also used to monitor reaction progress; the loss of phosphate signal at −1 ppm representing PEP showed reaction completion. Once the reaction had reached completion, the resulting mixture was analyzed by ESI–MS for the presence of Kdn-9-P mass (HRMS, ESI negative: calculated: 347.0385, found: 347.0394). A range of other sugars were screened as substrates using the same methodology. All sugar substrates were purchased from Sigma–Aldrich or Carbosynth. To establish kinetic parameters, a colorimetric phosphate-release assay was employed (BioMol Green, Enzo). This assay followed reaction progress by monitoring release of the by-product of the reaction, P_i_. The reactions mixtures included a fixed concentration of one substrate (100 μm) while varying the other (3.125, 6.25, 12.5, 25, 50, 100, and 200 μm) in a buffered solution containing 50 mm HEPES (pH 7.5), 2 mm MgCl_2_, and CAMPEP_0191217894 (final concentration, 4.7 μg/ml). The steady state kinetic values determined at 25 °C using this method are *k*_cat_ = 14.9 min^−1^, *V*_max_ = 30.4 ± 1.9 pmol min^−1^, ^(PEP)^*K_m_* = 18.3 ± 1.8 μm (at 100 μm Man-6-P), and ^(Man-6-P)^*K_m_* = 63.6 ± 10.5 μm (at 100 μm PEP) (Fig. 4*D*). The values and graphs were produced using GraphPad Prism (version 7).

A coupled assay was employed to confirm that the product of the Kdn-9-P synthetase enzyme was accepted by *B. thetaiotaomicron* VPI-5482 Kdn-9-P phosphatase. Reaction conditions were essentially the same as described previously, but an excess of PEP was used with respect to Man-6-P (10 and 8 mm, respectively). A no-enzyme ^31^P NMR time point was acquired before 38 μg of CAMPEP_0191217894 (Kdn-9-P synthetase) was added. The reaction was left for 3 h at which point formation of Kdn-9-P and P_i_ was observed (Fig. 4*C*). Kdn-9-P phosphatase from *B. thetaiotaomicron* VPI-5482 was then added to the reaction mixture (4 μg), and the reaction was allowed to progress a further 1 h.

For analysis of CMP–Kdn synthetase, reaction mixtures contained 20 μg of CAMPEP_0191219004, 10 mm MgCl_2_, 8 mm of CTP, and 10 mm of commercial Kdn or Neu5Ac (Sigma–Aldrich) buffered in 100 mm Tris-HCl (pH 9). A ^31^P NMR no-enzyme time point was taken before addition of CAMPEP_0191219004. The reaction reached completion when signals for CTP (−5.83 ppm, doublet, −10.75 ppm, doublet, −19.38 ppm, triplet) had diminished. At this point, CMP–Kdn was purified using strong anion exchange as outlined in Wagstaff *et al.* (64). ^1^H NMR, ^31^P NMR, and MS values aligned well with previously described literature (21, 65) (Fig. S4 and S5); ^1^H NMR (D_2_O) ppm: 7.9 (d, *J* = 7.5 Hz, 1H), 6.04 (d, *J* = 7.5 Hz, 1H), 5.91 (d, *J* = 4.5 Hz, 1H), 4.27 (m, 1H), 4.22 (t, *J* = 4.8 Hz, 1H), 4.16 (d, *J* = 5.3 Hz, 3H), 4.01 (dd, *J* = 10 Hz, J = 1 Hz, 1H), 3.95 (ddd, *J* = 11.5 Hz, *J* = 9.4 Hz, *J* = 4.9 Hz, 1H), 3.85 (m, 2H), 3.72–3.45 (m, 16H, buffer protons and H-5,7,8 of Kdn), 2.36 (dd, *J* = 13 Hz, 1H), 1.52 (ddd, *J* = 14 Hz, *J* = 13.5 Hz, *J* = 5.7, 1H); ^31^P NMR (H_2_O) ppm: −4.61 (s) (HRMS, ESI negative: calculated: 572.1129, found: 572.1135).

### Bioinformatic analysis

Kdn-9-P synthetase (CAMPEP_0191217894) and CMP–Kdn synthetase (CAMPEP_0191219004) protein sequences from *P. parvum* Texoma1 were used as consensus sequences in BLASTp analysis of other algal transcriptomes from the MMETSP database (37). The green alga, *P. parkae*, was found to have low sequence identity homologs to both *Prymnesium* transcripts, instead displaying high sequence similarity to a characterized Kdo-8-P synthetase from *Haemophilus influenzae* and CMP-Kdo synthetase from *Pseudomonas aeruginosa* (44% sequence identity, Protein Data Bank code 1O60; and 41% sequence identity, Protein Data Bank code 4XWI, respectively). These new transcripts were used in addition to *P. parvum*'s to query a total of 153 nucleic acid databases from other algal groups. A full list of databases used can be found in Table S2. Sequences displaying *E* values ≤ 1E^−10^ were subject to manual inspection of conserved domains before being assigned as a hit.

For phylogenetic analysis, two independent sets of algal sequences identified in BLASTp analysis (sialic acid synthetase-like sequences and CMP–Kdn synthetase sequences) were aligned using the default settings of MAFFT (54). Maximum-likelihood phylogenetic trees were constructed using MEGA7 (55) and drawn using iTOL (56). Bootstrap values were based on 100 resampling permutations, and branches with values >50% are labeled on the trees with a *black circle*.

## Author contributions

B. A. W. and R. A. F. conceptualization; B. A. W. data curation; B. A. W. and M. R. formal analysis; B. A. W. and M. R. investigation; B. A. W. and M. R. methodology; B. A. W. writing-original draft; B. A. W., M. R., and R. A. F. writing-review and editing; M. R. and R. A. F. supervision; R. A. F. resources; R. A. F. funding acquisition; R. A. F. project administration.

## Supplementary Material

Supporting Information

## Footnotes

3 Please note that the JBC is not responsible for the long-term archiving and maintenance of this site or any other third party hosted site.

## Footnotes

2 The abbreviations used are:

Kdn: 3-deoxy-d-*glycero*-d-*galacto*-2-nonulosonic acid
P: phosphate
MMETSP: Marine Microbial Eukaryote Transcriptome Sequencing Project
DMB: 1,2-diamino-4,5-methylenedioxybenzene
ESI: electrospray ionization
PEP: phosphoenolpyruvate
Kdo: 3-deoxy-d-*manno*-2-octulosonic acid
HRMS: high resolution mass spectrometry.
