# Supplementary material for: Identification of a Kdn biosynthesis pathway in the haptophyte Prymnesium parvum suggests widespread sialic acid biosynthesis among microalgae
Source: J Biol Chem. 2018 Aug 31;293(42):16277–90. doi: 10.1074/jbc.RA118.004921 (PMC6200933; doi:10.1074/jbc.RA118.004921)
Supplement: Supporting Information [file supp_293_42_16277__index.html]

Identification of a Kdn biosynthesis pathway in the haptophyte Prymnesium parvum suggests widespread sialic acid biosynthesis among microalgae — Kdn biosynthesis in microalgae — Identification of a Kdn biosynthesis pathway in the haptophyte Prymnesium parvum suggests widespread sialic acid biosynthesis among microalgae — Kdn biosynthesis in microalgae — Supporting Information 

# Identification of a Kdn biosynthesis pathway in the haptophyte *Prymnesium parvum* suggests widespread sialic acid biosynthesis among microalgae

## Supporting Information

- Supporting Information (to be published online) - Revised Version 2
- Table S2 - Supplementary Table 2 detailing a full list of sequences examined in this study, their database origin, sequence identifiers and organism they were identified from
