# Supplementary material for: Identification of a Kdn biosynthesis pathway in the haptophyte Prymnesium parvum suggests widespread sialic acid biosynthesis among microalgae
Source: J Biol Chem. 2018 Aug 31;293(42):16277–90. doi: 10.1074/jbc.RA118.004921 (PMC6200933; doi:10.1074/jbc.RA118.004921)
Supplement: Supporting Information [file supp_RA118.004921_139489_1_supp_194309_p8b828.pdf]

Supplementary Information

Identification of a Kdn biosynthesis pathway in the haptophyte *Prymnesium parvum* suggests widespread sialic acid biosynthesis among microalgae

Ben A. Wagstaff[1], Martin Rejzek[1] and Robert A. Field[1]

From the [1]Department of Biological Chemistry, John Innes Centre, Norwich Research Park, Norwich, NR4 7UH, United Kingdom

Running title: Kdn biosynthesis in microalgae

*To whom correspondence should be addressed: Robert A. Field: Department of Biological Chemistry, John Innes Centre, Norwich Research Park, Norwich, Norfolk, NR4 7UH, UK; rob.field@jic.ac.uk; tel. (+44)-1603-450720

Contents

Table S1 – Strains and species of the *Prymnesium* genus used in this study. 2

Figure S1 – Multiple strains of the *Prymnesium* genus contain Kdn. 3

Figure S2 – Sugar nucleotide profiling shows *P. parvum* 946/6 contains CMP-Kdn. 4

Figure S3 – Recombinant expression of *P. parvum* Kdn-9-P synthetase, *B. thetaiotamicron* Kdn-9-P phosphatase and *P. parvum* CMP-Kdn synthetase in *E. coli*. 5

Figure S4 – ^{1}H NMR of CMP-Kdn produced in this study. 6

Figure S5 - Expanded (3.4 – 4.4 ppm) ^{1}H NMR spectrum from Figure S4 of CMP-Kdn produced in this study. 7

SEQUENCES USED FOR PROTEIN EXPRESSION 8

Genus/Species	Strain Identifier	Source
Prymnesium parvum	946/6	Culture Collection of Algae and Protozoa (CCAP)
Prymnesium parvum	94A	Marine Biological Association Culture Collection
Prymnesium parvum	94C	“
Prymnesium parvum	579	“
Prymnesium patelliferum	527A	“
Prymnesium patelliferum	527C	“
Prymnesium patelliferum	527D	“
Prymnesium sp.	522	“
Prymnesium sp.	569	“
Prymnesium sp.	592	“
Prymnesium sp.	593	“
Prymnesium sp.	595	“
Prymnesium sp.	596	“
Prymnesium sp.	597	“
Prymnesium sp.	598	“

Table S1 – Strains and species of the *Prymnesium* genus used in this study.

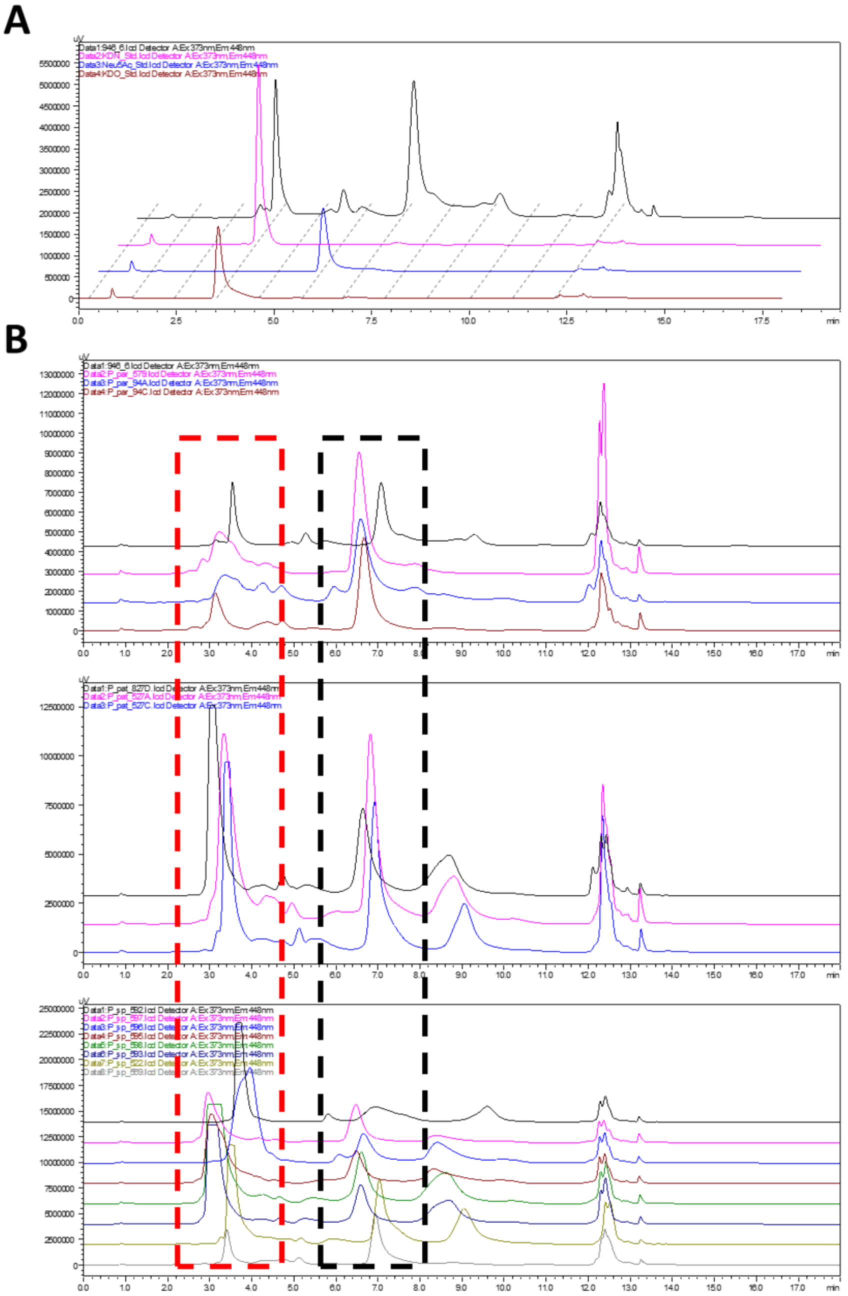

Figure S1 – Multiple strains of the *Prymnesium* genus contain Kdn. (A) *P. parvum* 946/6 contains peaks aligning with Kdn/Kdo and are confirmed to be Kdn by subsequent MS analysis (Figure 3C). Brown – Kdo standard. Blue – Neu5Ac standard. Pink – Kdn standard. (B) Top – 4 *P. parvum* strain extracts. Middle – 3 *P.* sp. strain extracts. Bottom – 8 *P. patelliferum* strain extracts. Peaks around 2.6-3.2 minutes (red hashed line) correspond to DMB-Kdn and are confirmed by identification of corresponding DMB-Kdn masses (Figure 3C). All other peaks (including those in black hashed lines) are unknown but do not relate to masses of any known DMB-sialic acid adducts or their acetylated derivatives.

A

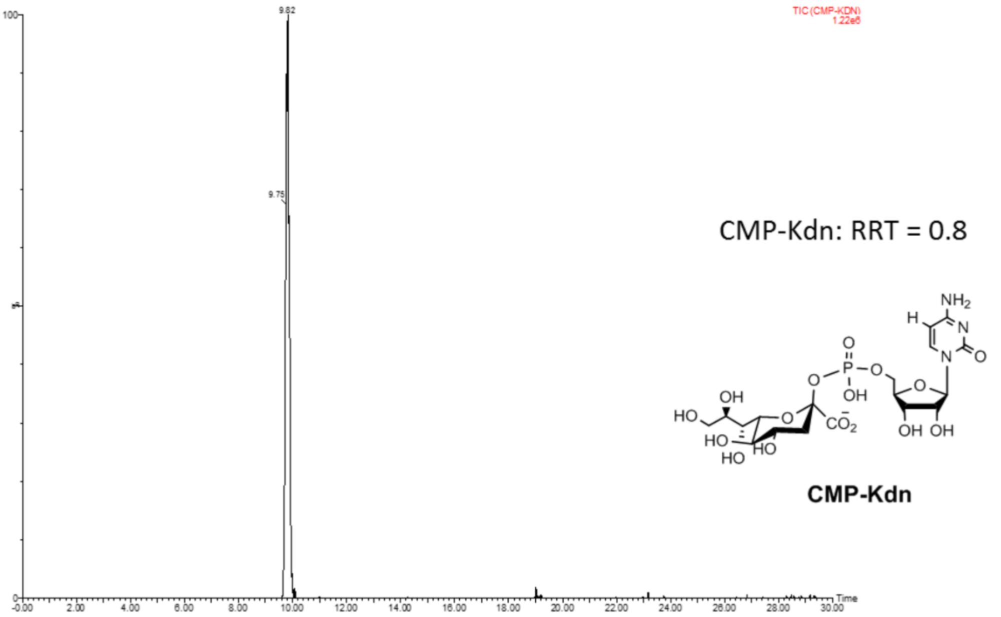

B

Sugar Nucleotide Species	Relative Retention Time [min]	MRM transitions	Fragment	Concentration [pmol/g of cell pellet]
CMP-β-D-Kdn	0.8	572 → 322<br>572 → 97	$[NMP\text{-}H]^-$<br>$[H_3PO_4\text{-}H]^-$	206.6 ± 72.1*
UDP-α-D-Glc	1	565 → 323<br>565 → 79	$[NMP\text{-}H]^-$<br>$[H_3PO_4\text{-}H_3O]^-$	328.2 ± 101.0*
UDP-α-D-Gal	0.92	565 → 323<br>565 → 159	$[NMP\text{-}H]^-$<br>$[H_4P_2O_7\text{-}H_3O]^-$	71.2 ± 17.4*
GDP-α-D-Glc (added internal standard)	1.56	604 → 362<br>604 → 241	$[NMP\text{-}H]^-$<br>$c[Glc\text{-}1\text{-}P\text{-}H\text{-}H_2O]^-$	1540.0**

Figure S2 – Sugar nucleotide profiling shows *P. parvum* 946/6 contains CMP-Kdn. (A) LC-MS chromatogram showing a strong signal for CMP-Kdn based on MRM transitions of authentic CMP-Kdn standard. (B) Sugar nucleotides examined in this study (CMP-β-D-Kdn) and others shown for comparison (UDP-α-D-Glc, UDP-α-D-Gal and internal standard GDP-α-D-Glc). *The data are mean of 3 biological replicates, ± indicates standard error. ** GDP-α-D-Glc (1540 pmol / g wet pellet) was added to enable quantification of other sugar nucleotides.

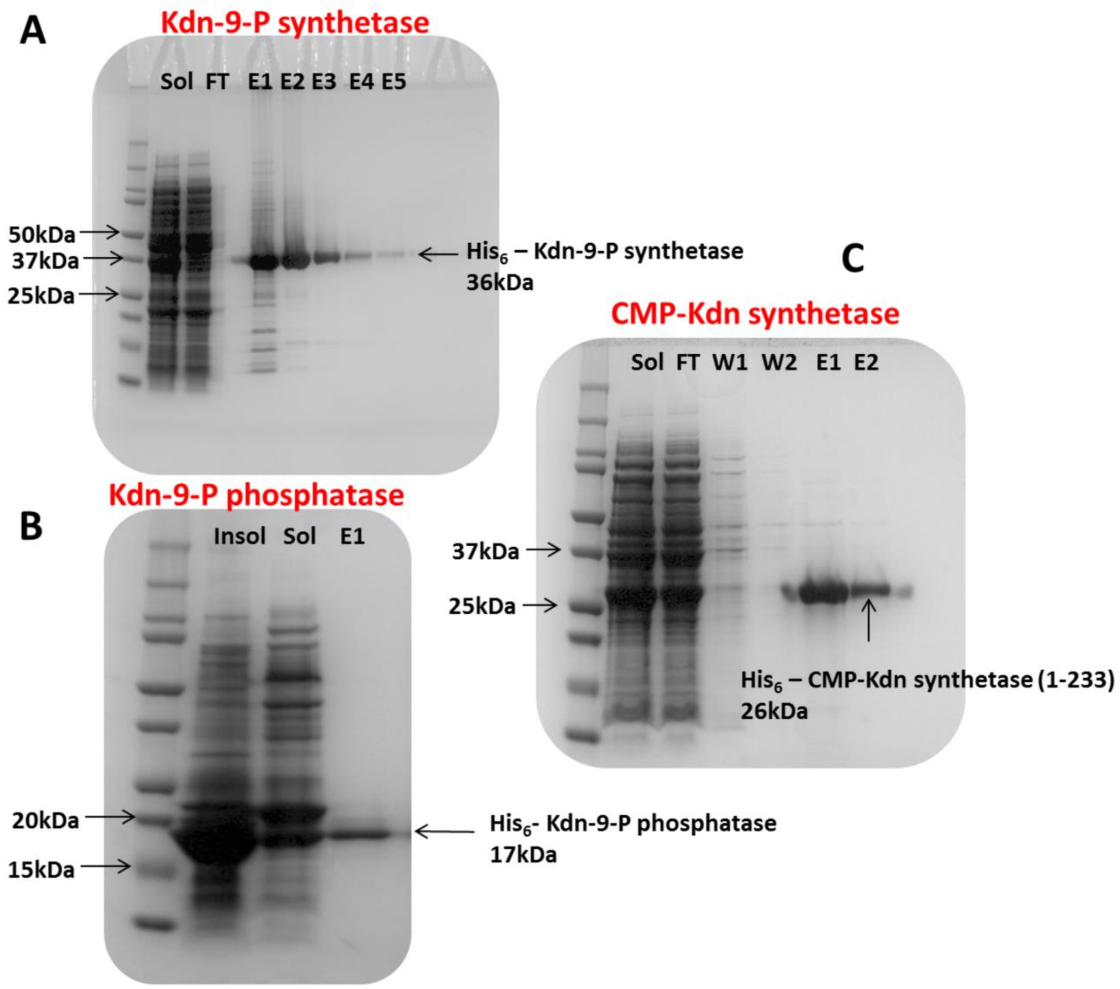

Figure S3 – Recombinant expression of *P. parvum* Kdn-9-P synthetase, *B. thetaiotamicron* Kdn-9-P phosphatase and *P. parvum* CMP-Kdn synthetase in *E. coli*. (A) SDS-PAGE analysis of recombinant *P. parvum* Kdn-9-P synthetase. (B) SDS-PAGE analysis of recombinant *B. thetaiotamicron* Kdn-9-P phosphatase. (C) SDS-PAGE analysis of recombinant *P. parvum* CMP-Kdn synthetase.

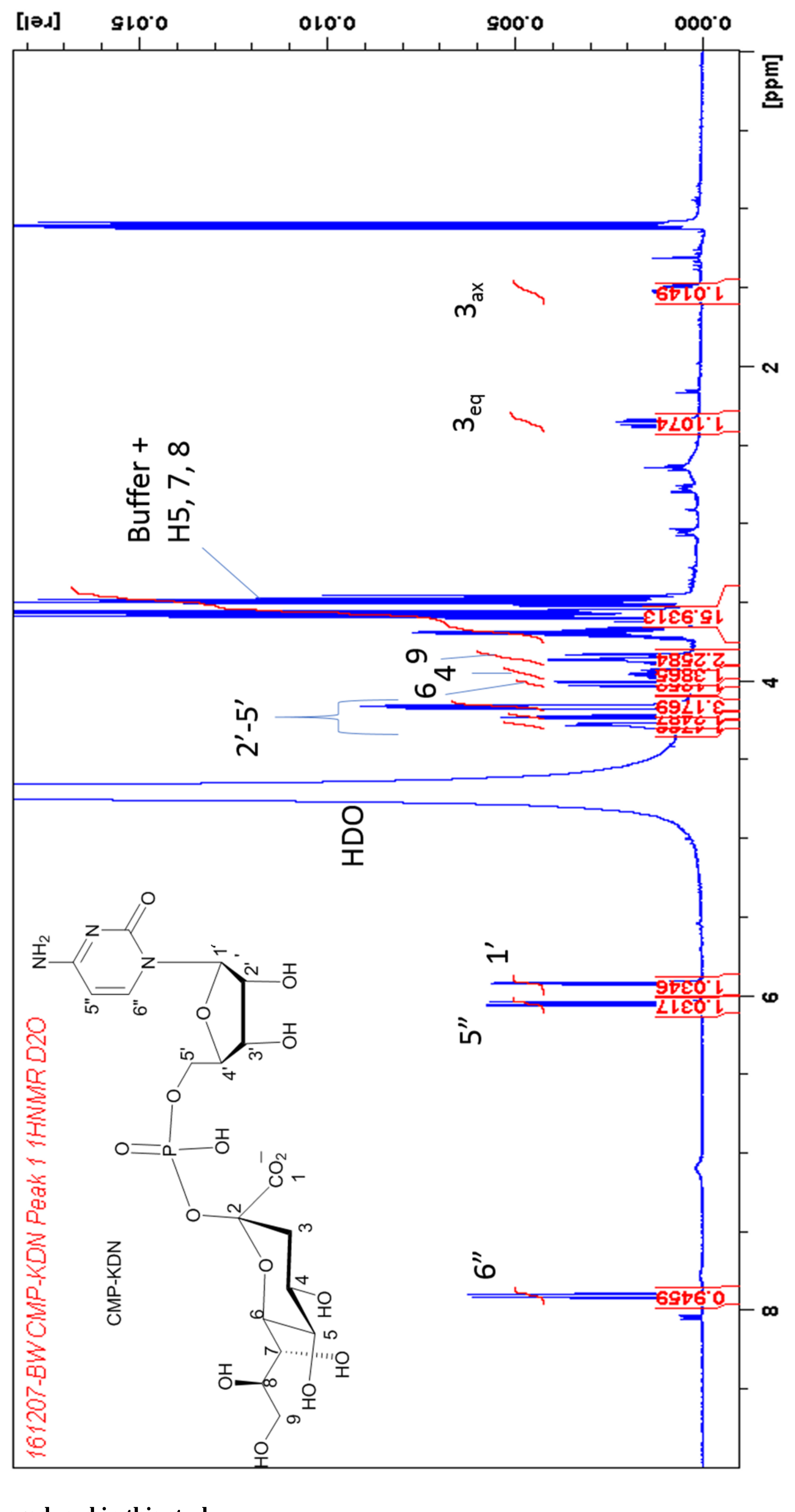

Figure S4 – ^{1}H NMR of CMP-Kdn produced in this study.

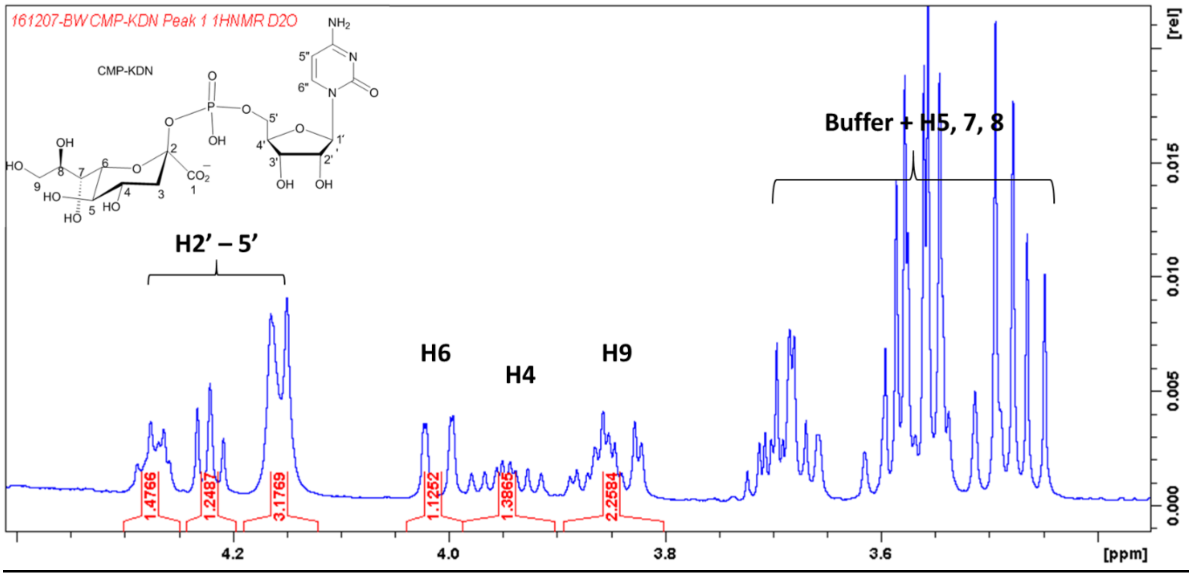

Figure S5 - Expanded (3.4 – 4.4 ppm) ^{1}H NMR spectrum from Figure S4 of CMP-Kdn produced in this study.

SEQUENCES USED FOR PROTEIN EXPRESSION

Sequences used in this study for protein expression. Underlined sequences are overhangs added to the protein sequences for direct cloning into pOPINF vectors using In-Fusion ™ cloning.

Codon optimized Kdn-9-P Synthetase – *E. coli*

ATG AGC GCC AAA AAA CAG AAA GTA GAC GCC GCT CCA GCG CCT ATT GTA TAT CAC GAA CCG AAA GTG ATG GCG GAG ATT GGA TGT AAC CAT ATG GGC GAT CTG GAA ATC GCA AAG GAG TTA CTG ACG CTG GCA AAA CAG GCG GGT GCA GAG TAT GGG AAA TTT CAG AAA CGG AAT CCA AAA GAA CTG CTT ACG GTG GAG CAG TAC GCG GCC CCA CAC CCG AAT CCG CGT AAT AGC TAT GGT GAT ACA TAT GGC GCG CAT CGC GAG TTT TTG GAG TTC ACT ATT GAG CAG CAT GCG GAA TTG AAG AAG CAC TGC GAG AAA ATT GGC TTA GGT TAC TCG TGT AGC GTA TGG GAC ATG ACT TCT GCG AAA GAA ATT GCG TCG ATT AAC CCG GAC CTG ATC AAA GTT GGC TCG CCC AGC AAC CAA CAT TGG GAG ATG CAG AAA ATC CTC CGT GAC GAA TAC AGC GGG GAC GTT CAC ATC TCC ACG GGT ATG ACT ACA AAA GAA GAA ATT GAG AAG ATC GTG CAA TTT TGG GAG GAG GGA AAA GGT GAT GCC AAA AAT CGG CTG GTA TTA TAT AAT TGC ACC AGC GGT TAT CCG GTC CCG TTT GAA GAT GTT TGC CTG CTG GAG CTC CGT GAA CTC CAC GCC CTG TAT GCT GGT CGC GTG AAG CAT TTA GGG TTC TCT GGG CAC CAC CTG GGT ATC GCC ATT GAT ATT GCA GCA TAT GCC CTC GGC GCC ACA TGG AAC GAA CGC CAT TTC ACC AAA GAT CGC ACT TGG AAA GGA ACA GAT CAT GCT GCG AGT CTG GAA CCA GCG GGC TTG AGC AAA CTG TGC CGT GAT CTG AAG GCG ACT TGG AAA TGC ATG AGC ACT AAG AAA ACC GAA ATC CTG CCG ATC GAA AGC GAG CAG CGC GCC AAG CTG AAA TGG GGT TGC TAT AAC GCT AGC AAA GTG GTG AAG TAA

Modified Kdn-9-P synthetase with pOPIN overhangs and no start/stop codon ordered from IDT gBlock service:

AAGTTCTGTTTCAGGGCCCG AGC GCC AAA AAA CAG AAA GTA GAC GCC GCT CCA GCG CCT ATT GTA TAT CAC GAA CCG AAA GTG ATG GCG GAG ATT GGA TGT AAC CAT ATG GGC GAT CTG GAA ATC GCA AAG GAG TTA CTG ACG CTG GCA AAA CAG GCG GGT GCA GAG TAT GGG AAA TTT CAG AAA CGG AAT CCA AAA GAA CTG CTT ACG GTG GAG CAG TAC GCG GCC CCA CAC CCG AAT CCG CGT AAT AGC TAT GGT GAT ACA TAT GGC GCG CAT CGC GAG TTT TTG GAG TTC ACT ATT GAG CAG CAT GCG GAA TTG AAG AAG CAC TGC GAG AAA ATT GGC TTA GGT TAC TCG TGT AGC GTA TGG GAC ATG ACT TCT GCG AAA GAA ATT GCG TCG ATT AAC CCG GAC CTG ATC AAA GTT GGC TCG CCC AGC AAC CAA CAT TGG GAG ATG CAG AAA ATC CTC CGT GAC GAA TAC AGC GGG GAC GTT CAC ATC TCC ACG GGT ATG ACT ACA AAA GAA GAA ATT GAG AAG ATC GTG CAA TTT TGG GAG GAG GGA AAA GGT GAT GCC AAA AAT CGG CTG GTA TTA TAT AAT TGC ACC AGC GGT TAT CCG GTC CCG TTT GAA GAT GTT TGC CTG CTG GAG CTC CGT GAA CTC CAC GCC CTG TAT GCT GGT CGC GTG AAG CAT TTA GGG TTC TCT GGG CAC CAC CTG GGT ATC GCC ATT GAT ATT GCA GCA TAT GCC CTC GGC GCC ACA TGG AAC GAA CGC CAT TTC ACC AAA GAT CGC ACT TGG AAA GGA ACA GAT CAT GCT GCG AGT CTG GAA CCA GCG GGC TTG AGC AAA CTG TGC CGT GAT CTG AAG GCG ACT TGG AAA TGC ATG AGC ACT AAG AAA ACC GAA ATC CTG CCG ATC GAA AGC GAG CAG CGC GCC AAG CTG AAA TGG GGT TGC TAT AAC GCT AGC AAA GTG GTG AAG TAAAGCTTTCTAGACCAT

Codon optimized Kdn-9-P phosphatase – *E. coli*

ATGAAAGAAA TCAAATTGAT TCTGACCGAC ATCGATGGTG TTTGGACGGA CGGTGGAATG TTCTACGATC AGACGGGTAA CGAATGGAAA AAATTTAACA CTTCTGATTC TGCCGGTATT TTCTGGGCAC ATAACAAAGG AATTCCGGTG GGCATCCTGA CCGGAGAAAA GACGGAAATC GTCCGTCGCC GTGCGGAAAA ACTGAAGGTC GATTATCTGT TCCAAGGTGT AGTTGATAAA TTATCAGCGG CCGAGGAACT GTGCAACGAG CTTGGTATCA ATTTGGAACA GGTCGCCTAC ATTGGTGATG ATTTAAACGA TGCCAAACTT TTGAAACGCG TGGGTATCGC TGGTGTACCT GCGTCAGCGC CCTTCTACAT TCGTCGCCTG TCAACGATCT TTTTGGAAAA ACGGGGCGGC GAAGGTGTGT TTCGCGAATT TGTTGAAAAA GTTCTGGGTA TCAATCTGGA GGATTTTATT GCTGTCATCC AATG

Modified Kdn-9-P phosphatase with pOPIN overhangs and no start/stop codon:

AAGTTCTGTTTCAGGGCCCG AAAGAAA TCAAATTGAT TCTGACCGAC ATCGATGGTG TTTGGACGGA CGGTGGAATG TTCTACGATC AGACGGGTAA CGAATGGAAA AAATTTAACA CTTCTGATTC TGCCGGTATT TTCTGGGCAC ATAACAAAGG AATTCCGGTG GGCATCCTGA CCGGAGAAAA GACGGAAATC GTCCGTCGCC GTGCGGAAAA ACTGAAGGTC GATTATCTGT TCCAAGGTGT AGTTGATAAA TTATCAGCGG CCGAGGAACT GTGCAACGAG CTTGGTATCA ATTTGGAACA GGTCGCCTAC ATTGGTGATG ATTTAAACGA TGCCAAACTT TTGAAACGCG TGGGTATCGC TGGTGTACCT GCGTCAGCGC CCTTCTACAT TCGTCGCCTG TCAACGATCT TTTTGGAAAA ACGGGGCGGC GAAGGTGTGT TTCGCGAATT TGTTGAAAAA GTTCTGGGTA TCAATCTGGA GGATTTTATT GCTGTCATCC AA TAAAGCTTTCTAGACCAT

Codon optimized CMP-Kdn synthetase – *E. coli*

ATG ACC GTT TGG CAT CCG GTA CCT GAG GTA CGT ATT GTA GCG GTA ATT CCG GCA CGT GGT GGC AGC GTT TCG ATT CCC CGG AAA AAC ATT AAG CCT CTG GCG GGC CGC CCG CTG ATC GAT TGG GTC ATC AAA CCG GCG CTG CAC TGC GGG ATT TTT ACC GAT GTA TAC GTG AGC ACC GAC GAT GAT GCT ATC GCG AGC GTC GCT GAA AAA TGT GGC GCC AAA GTG CAT CGG CGT GAT CCG GCC ACG GCG ACC GCT ACG GCC ACC ACC GAG TCT GCG CTG CTT GAC TTC GCG CAG TCA CAC GGT GAC TTT GAC GTA CTG TGT CTT ATT CAA GCA ACC TCC CCG TTT ATT ACC CCT CGC GAT CTG ATT AAC GGC TGG GAA TTA ATG CGC GCC ATG GAA GCC GAT AGC CTC GTA ACC GCG GTG CGT GCG CAT CGC TTC CTT TGG CAG GTT GAC AAA GAT ACA GGT CTT GCG AAA GCG AAA AAC TAT GAC CCA CTG AAA CGC CCG CGC CGT CAG GAC TGG GAT GGG GAA CTG GTG GAG AAT GGC GCT TTT TAC ATG ACC ACC AAA GCA TGC TTA GAG AAA CAT AAA TGT CGC CTC GGG GAA AAG ATG GTC CTG CTG GAG ATG GAA GAG CAT ACG TTT ACT GAA CTG GAT TCG TTA GTA GAC TGG CAG ATC GTG ACC AAT ATG ACC GAA AAT TAC GGT TAC TGG CCG CCG CGT AAC TGG GGT GAA GCC GCG TCC TCC TCA GCC CGT CCG GAC GCG GCC AAG ATC GTA TTG TGT GCG CTG GGC GTT CTG GCT CTG GGT CTG TCG ATT GGA CGT ATG AGC AAA TAA

Modified CMP-Kdn synthetase with pOPIN overhangs and no start/stop codon:

AAGTTCTGTTTCAGGGCCCG ACC GTT TGG CAT CCG GTA CCT GAG GTA CGT ATT GTA GCG GTA ATT CCG GCA CGT GGT GGC AGC GTT TCG ATT CCC CGG AAA AAC ATT AAG CCT CTG GCG GGC CGC CCG CTG ATC GAT TGG GTC ATC AAA CCG GCG CTG CAC TGC GGG ATT TTT ACC GAT GTA TAC GTG AGC ACC GAC GAT GAT GCT ATC GCG AGC GTC GCT GAA AAA TGT GGC GCC AAA GTG CAT CGG CGT GAT CCG GCC ACG GCG ACC GCT ACG GCC ACC ACC GAG TCT GCG CTG CTT GAC TTC GCG CAG TCA CAC GGT GAC TTT GAC GTA CTG TGT CTT ATT CAA GCA ACC TCC CCG TTT ATT ACC CCT CGC GAT CTG ATT AAC GGC TGG GAA TTA ATG CGC GCC ATG GAA GCC GAT AGC CTC GTA ACC GCG GTG CGT GCG CAT CGC TTC CTT TGG CAG GTT GAC AAA GAT ACA GGT CTT GCG AAA GCG AAA AAC TAT GAC CCA CTG AAA CGC CCG CGC CGT CAG GAC TGG GAT GGG GAA CTG GTG GAG AAT GGC GCT TTT TAC ATG ACC ACC AAA GCA TGC TTA GAG AAA CAT AAA TGT CGC CTC GGG GAA AAG ATG GTC CTG CTG GAG ATG GAA GAG CAT ACG TTT ACT GAA CTG GAT TCG TTA GTA GAC TGG CAG ATC GTG ACC AAT ATG ACC GAA AAT TAC GGT TAC TGG CCG CCG CGT AAC TGG GGT GAA GCC GCG TCC TCC TCA GCC CGT CCG GAC GCG GCC AAG ATC GTA TTG TGT GCG CTG GGC GTT CTG GCT CTG GGT CTG TCG ATT GGA CGT ATG AGC AAATAAAGCTTTCTAGACCAT

Modified truncated CMP-Kdn synthetase with pOPIN overhangs and no start/stop codon ordered from IDT gBlock service:

AAGTTCTGTTTCAGGGCCCG ACC GTT TGG CAT CCG GTA CCT GAG GTA CGT ATT GTA GCG GTA ATT CCG GCA CGT GGT GGC AGC GTT TCG ATT CCC CGG AAA AAC ATT AAG CCT CTG GCG GGC CGC CCG CTG ATC GAT TGG GTC ATC AAA CCG GCG CTG CAC TGC GGG ATT TTT ACC GAT GTA TAC GTG AGC ACC GAC GAT GAT GCT ATC GCG AGC GTC GCT GAA AAA TGT GGC GCC AAA GTG CAT CGG CGT GAT CCG GCC ACG GCG ACC GCT ACG GCC ACC ACC GAG TCT GCG CTG CTT GAC TTC GCG CAG TCA CAC GGT

GAC TTT GAC GTA CTG TGT CTT ATT CAA GCA ACC TCC CCG TTT ATT ACC CCT CGC GAT CTG ATT AAC GGC TGG GAA TTA ATG CGC GCC ATG GAA GCC GAT AGC CTC GTA ACC GCG GTG CGT GCG CAT CGC TTC CTT TGG CAG GTT GAC AAA GAT ACA GGT CTT GCG AAA GCG AAA AAC TAT GAC CCA CTG AAA CGC CCG CGC CGT CAG GAC TGG GAT GGG GAA CTG GTG GAG AAT GGC GCT TTT TAC ATG ACC ACC AAA GCA TGC TTA GAG AAA CAT AAA TGT CGC CTC GGG GAA AAG ATG GTC CTG CTG GAG ATG GAA GAG CAT ACG TTT ACT GAA CTG GAT TCG TTA GTA GAC TGG CAG ATC GTG ACC AAT ATG ACC GAA AAT TAC GGT TAC TGGTAAAGCTTTCTAGACCAT
